# The effects of impulsivity and proactive inhibition on reactive inhibition and the go process: insights from vocal and manual stop signal tasks

**DOI:** 10.3389/fnhum.2015.00529

**Published:** 2015-10-06

**Authors:** Leidy J. Castro-Meneses, Blake W. Johnson, Paul F. Sowman

**Affiliations:** ^1^Department of Cognitive Science, Australian Research Council Centre of Excellence in Cognition and its Disorders, Macquarie UniversityNorth Ryde, NSW, Australia; ^2^Department of Cognitive Science, Perception in Action Research Centre, Macquarie UniversityNorth Ryde, NSW, Australia

**Keywords:** vocal inhibition, selective inhibition, response inhibition, reactive inhibition, proactive inhibition, impulsivity, dysfunctional impulsivity

## Abstract

This study measured proactive and reactive response inhibition and their relationships with self-reported impulsivity. We examined the domains of both vocal and manual responding using a stop signal task (SST) with two stop probabilities: high and low probability stop (1/3 and 1/6 stops respectively). Our aim was to evaluate the effect stop probability would have on reactive and proactive inhibition. We tested 44 subjects and found that for the high compared to low probability stop signal condition, more proactive inhibition was evident and this was correlated with a reduction in the stop signal reaction time (SSRT). We found that reactive inhibition had a positive relationship with dysfunctional but not functional impulsivity in both vocal and manual domains of responding. These findings support the hypothesis that proactive inhibition may pre-activate the network for reactive inhibition.

## Introduction

This study measured response inhibition via the stop signal paradigm (Vince, 1948; Lappin and Eriksen, 1966; Logan and Cowan, 1984) in two effector systems: vocal and manual. Response inhibition is described as the ability to stop a prepotent response (Logan and Cowan, 1984; Logan, 1994), which, in our study was either a spoken word or a button press. We measured two types of response inhibition: proactive and reactive inhibition. Proactive inhibition is defined as the advanced preparation to halt action in the anticipation of an imminent stop signal. Reactive inhibition is defined as the performance of outright stopping in response to the appearance of a stop signal (Chambers et al., 2009; Aron, 2011).

The analysis of the stop signal task (SST) is based on the horse race model proposed by Logan and Cowan (1984). The model assumes that the stop and the go processes are independent of each other in the sense that whichever finishes first, wins. This assumption is based on the fact that failed stop trials always have faster mean reaction times (RTs) compared to go trials, suggesting that participants fail to stop because the go process finishes before the stop process. For a model that proposes the go and stop processes interact, see Boucher et al. (2007). The horse race model was mainly developed by testing reactive inhibition (measured by the stop signal RT or SSRT) but has been little tested in the context of proactive inhibition manipulations (measured by the increment of go RTs in the context of possible stop signal appearance). Some evidence suggests that the complexity of go and stop tasks affect the latency of go and stop RTs. For example, regarding the complexity of the go task: go RTs are always slower in two or more choice-RT tasks than in simple RT tasks. Likewise the SSRT is longer when the go imperative consists of a choice RT task compared to a simple RT task (Logan et al., 1984; Riegler, 1986). Such observations suggest that the complexity of the go task interferes with both go and stop processes. Regarding the complexity of the stop task, some studies have increased the stop signal from one to two and asked participants to stop to one but to ignore the other stop signal (this refers to selective inhibition; Logan et al., 1986; Riegler, 1986). These studies find that go RTs are slower and SSRTs longer in selective inhibition tasks compared to simple inhibition tasks, suggesting that the complexity of the stop task interacts with both go and stop processes. In sum, the complexity of the go and the stop task interfere with each other. When either the go or stop task is complex, go RTs become slower and SSRTs become longer.

We interpreted these results to indicate that the complexity of the go and stop tasks added an additional variable: a slowing effect, which would probably help to perform either task more effectively. In particular, selective inhibition creates the need to hold the prepotent response more strongly because the stopping process is more complicated, requiring an increase in proactive inhibition. In other words, our idea was that in selective inhibition, proactive inhibition is increased to help reactive inhibition. In fact, this idea has already been supported by Chikazoe et al. (2009) and in the first experiment of Jahfari et al. (2010) who reported a significant negative relationship between proactive inhibition and the SSRT, a result which suggests that a greater level of preparation is related to faster reactive stopping. It has also been suggested that, proactive inhibition pre-activates the same inhibitory network for reactive inhibition and this is why participants are able to stop quickly, because the inhibitory network has been primed.

However, studies of the relationship between proactive and reactive inhibition have had mixed results: a third experiment reported in Jahfari et al. (2010) showed this relationship did not exist. The authors did not offer an explanation. We propose that the lack of a relationship could have been due to the amount of proactive inhibition that was used in these tasks: while in their first experiment proactive inhibition was measured as a slowing of go RT of 111.3 ms for the relevant compared to an irrelevant stop condition, in the third experiment this difference was only 55 ms. To investigate this idea, this study was designed to manipulate the level of proactive inhibition and assess the relationship between proactive and reactive inhibition. One way to manipulate the level of proactive inhibition is to manipulate stop probabilities. However, previous studies that have manipulated stop probability have shown that either there were no differences in the SSRT (Ramautar et al., 2004; Lansbergen et al., 2007) or they did not analyse the SSRT (Logan and Burkell, 1986). The lack of difference in SSRT could have been because the high probability stop in Ramautar et al. (2004) contained 1/2 stop and 1/2 go trials, which may not have been enough to induce significant proactive inhibition; on the other hand, Lansbergen et al. (2007) recruited participants with the lowest and highest scores on impulsivity, which may have influenced the lack of differences across stop probabilities.

Deficiencies in reactive inhibition have been related to speech disorders such as developmental stuttering (Eggers et al., 2013), Tourette syndrome (Ziemann et al., 1997), attention-deficit hyperactivity disorder (ADHD; Barkley, 1997; Rubia et al., 2001a; Aron and Poldrack, 2005), schizophrenia (Kiehl et al., 2000; Enticott et al., 2008), obsessive-compulsive disorder (OCD) and trichotillomania (Menzies et al., 2007; Penadés et al., 2007; Bohne et al., 2008) and adolescents at risk of alcoholism and other substance use (Nigg et al., 2006). Interestingly, studies have shown that differences in response inhibition can be related to the level of self-reported impulsivity in control subjects (Logan et al., 1997; van den Wildenberg and Christoffels, 2010).

Studies investigating impulsivity and reactive inhibition have reported, for example, slower manual reactive inhibition (i.e., SSRT from manual responses) in individuals with high relative to low impulsivity scores (Logan et al., 1997; Marsh et al., 2002; Farr et al., 2012) but others have failed to find such differences (Avila and Parcet, 2001; Rodrìguez-Fornells et al., 2002; Lijffijt et al., 2004; Lansbergen et al., 2007). Moreover, evidence suggests a positive relationship existed between reactive inhibition and impulsivity, which in turn suggests that longer SSRTs are associated with higher impulsivity scores. This positive relationship has been described in the manual effector system only (Logan et al., 1997). In a more recent study, van den Wildenberg and Christoffels (2010) found that longer SSRT were related with dysfunctional impulsivity (not functional impulsivity) for vocal responses (not manual responses). This study is very interesting because it teased apart two types of impulsivity: dysfunctional impulsivity, described as rapid reactions with a less adaptive approach (Dickman, 1990) and functional impulsivity, characterized as rapid responses in situations where this is more optimal (a more adaptive approach). Having a relationship between reactive inhibition and dysfunctional impulsivity is consistent with neuropsychological disorders where impulsive behaviors are inappropriate and less adaptive (Barkley, 1997; Kiehl et al., 2000; Rubia et al., 2001a; Aron and Poldrack, 2005; Menzies et al., 2007; Penadés et al., 2007; Bohne et al., 2008; Enticott et al., 2008). Although, the evidence from manual response inhibition studies suggests that impulsivity is also related to longer SSRTs (Logan et al., 1997), van den Wildenberg and Christoffels (2010) only found that dysfunctional impulsivity was related with vocal responses (not manual responses), possibly because of the relatively small sample size (14 participants).

In sum, there is evidence that suggests a greater level of proactive inhibition enhanced reactive inhibition, but one out of three experiments did not show this relationship. It is not clear why. Second, two studies have shown that manual response inhibition is related to impulsivity but two other studies fail to confirm this. More recent evidence has used a impulsivity scale that distinguishes between dysfunctional and functional impulsivity and found that high score in dysfunctional impulsivity is related to slower SSRTs with vocal not manual responses, finding that would contradict a previous study that have shown manual responses are related to impulsivity. In other to clarify these inconsistencies, our aims were to investigate across two response modalities (vocal and manual) the relationship between proactive and reactive inhibition, and the relationship between dysfunctional impulsivity and reactive inhibition in both manual and vocal responses. We developed an SST with two certainty conditions (certain and uncertain, similar to Chikazoe et al., 2009). While the certain conditions only had go trials, the uncertain conditions contained both go and stop trials. We manipulated proactive inhibition in the uncertain conditions by implementing two stop probability conditions: high and low (similar to Logan and Burkell, 1986; Ramautar et al., 2004; Lansbergen et al., 2007). The high probability stop condition consisted of 1/3 stops and 2/3 go trials; the opposite was the case for the low probability stop condition, which was comprised of 1/6 stops and 5/6 go trials. We predicted that the high probability stop condition would induce more proactive inhibition relative to the low probability stop condition. We also predicted that the high level of proactive inhibition in the high probability stop would make SSRTs shorter. We hypothesized that if high, relative to low probability stops required more proactive inhibition, then the SSRT would be reduced for the high probability stopping. Further, we hypothesized that there would be a positive relationship between proactive and reactive inhibition. Our final aim was to re-investigate the relationship between dysfunctional impulsivity and reactive inhibition in both effector systems; our prediction was that manual responses would also be related to dysfunctional impulsivity. We hypothesized that the SSRT of both vocal and manual responses would be positively correlated with dysfunctional impulsivity.

## Methods

### Participants

Forty-six participants completed this study. Two participants were excluded because they did not meet the SST performance criteria of successfully stopping on ~50% of stop trials; one of these subjects progressively slowed throughout the experiment on the uncertain go trials and thus, this person was able to stop on 96% of the stops (p_inhibit = 0.96). The second person did the opposite and did not stop appropriately at the stop-signal, returning a percentage of unsuccessful stopping of 23% (p_respond = 0.23). Data analysis was performed on the remaining 44 participants (age range = 18–29; mean age = 20.5 years; *SD* = 2.73; 8 males). All participants had normal or corrected-to-normal vision, and reported no history of neurological impairment or psychiatric illness. All participants provided written informed consent. The study was approved by Macquarie University Human Research Ethics Committee.

### Apparatus

The experimental task was controlled in Presentation® software (version 16.1, www.neurobs.com) and was delivered via Samsung monitor (SyncMaster SA950_LS27A950, 27 inches, 1920 × 1080 pixels, 120 Hz refresh rate). Vocal-responses were sample at 48 kHz via an external microphone placed within 2 cm from each subject's mouth. Manual-responses involved a key press on a button box; Participants were seated approximately at 80 cm from the monitor.

### Stop signal task

**Figure 1:**
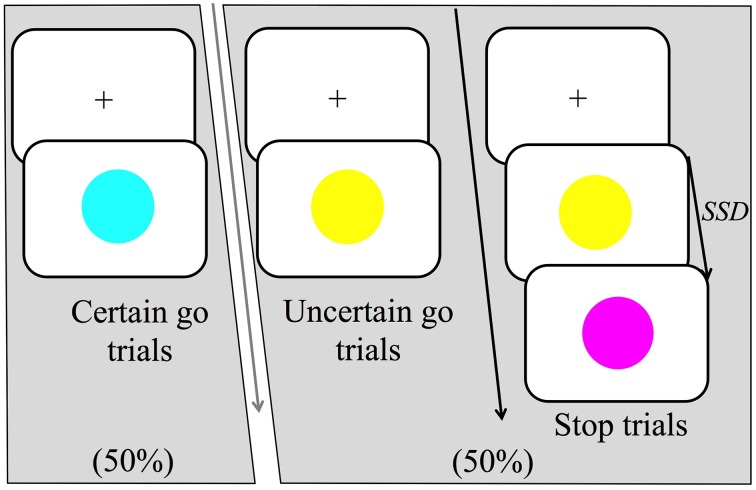
**Trial structure of the stop-signal task (SST)**. There were three main trial types: certain go, uncertain go and stop trials. Certain go trials were signaled by a blue circle and always required to either press a response button (manual-responses) or produce the short vowel sound “ɪ” as it would occur in the word “hit/hɪt/”(vocal-responses). Uncertain go trials were signaled by a yellow circle and required a response as in the certain go trials. Finally, stop trials started as uncertain go trials but after the stop signal delay (SSD), a stop-signal was presented, which was signaled by a purple circle. Participants were instructed to attempt to withhold their responses on seeing the stop signal. In the high probability stop condition, stop signals occurred following 2/3 of the uncertain go signals whereas in the low probability stop condition stop signals occurred following 1/3 of the uncertain go signals.

This study implemented a variant of the stop-signal task or SST (Logan and Cowan, 1984; Logan, 1994). It contained three types of trials (certain go, uncertain go, and stop) that all occurred within every block. All trials began with a black fixation cross appearing in the center of a white background; the duration of fixation randomly varied between 1 and 2.5 s. Certain go trials consisted of a simple reaction time task where participants were required to respond as quickly as possible to the certain go-signal, which was indicated by the onset of a blue circle, 10.5 cm in diameter (see Figure 1) in the center of the monitor. Certain go trials made up 50% of the total trial number. For manual responses, participants were asked to press a response button as quickly as possible whereas for vocal responses, participants were asked to make the short vowel sound “ɪ” as it would occur in the word “hit/hɪt/.” The other half of the trials was uncertain go trials in which the onset of yellow circle was the signal to initiate a response. The uncertainty in this trial type was created by the possibility of a stop signal following the go signal (yellow circle). The stop signal could appear with a probability of either one third or two thirds of all uncertain go trials. Hereafter we refer to these as low probability and high probability stop signals respectively. Participants were required to respond to the yellow circle as if this was a blue circle unless the stop-signal appeared.

Stop trials appeared only after uncertain go signals and were represented by a purple circle. In response to the stop signal, participants were instructed to attempt to withhold any response they might have initiated. The time at which the stop-signal was presented is referred to as the stop-signal delay (SSD). The SSD changed dynamically throughout the experiment via a staircase method that depended on each participant's performance. If a participant inhibited successfully on a stop trial, then successful response inhibition was made less likely on the subsequent stop trial by increasing the SSD by 30 ms. Contrarily, if the participant failed to stop, successful response inhibition was made more likely for the following stop trial by decreasing the SSD by 30 ms. Two independently adjusted staircases were employed that started with a SSD of 200 ms. One staircase was for the low probability stop and the other for the high. The task also contained a warning buzz that sounded when a go response was given after 700 ms or when the SSD dropped to 130 ms.

Overall, there were 900 trials in each response modality (either manual or vocal), which were divided into 6 blocks of 150 trials. In each block, 75 trials were certain go trials (50%) and 75 were uncertain go trials (50%). For the high probability stop blocks, 50 of the uncertain go trials were stop trials whereas for the low probability stop blocks, 25 were stop trials. Each response modality was tested in separate blocks counterbalanced for order across subjects. We also counterbalanced the high and low probability stop blocks. To make these probability blocks comparable, we needed to have an equal number of stop trials for each probability of stopping, thus, out of the 6 blocks, two blocks corresponded to the high probability stop condition (50 stop trials ^*^ 2 blocks = 100 stops) and four blocks were for the low probability stops (25 stop trials ^*^ 4 blocks = 100 stops). Because the number of blocks for the low probability stops was double that of the high probability stops, we implemented the condition that there would always be two low probability stop blocks between each high probability stop block. Based on this, we implemented two types of overall block presentation order, which were the only possible permutations that meet the condition of having two low probability blocks between high probability blocks and which also allowed counterbalancing of the order of the first probability block type over subjects. The first order started with a high probability stop block, therefore, block 1 and 4 were high probability stops and blocks 2, 3, 5, and 6 were low probability stops. The second order started with a low probability stop block (blocks 1, 2, 4, and 5) and blocks 3 and 6 were high probability stops. There were instructions at the beginning of each block which communicated the probability of stopping that would follow, it could either say that a stop signal would occur on either one third or two thirds of the uncertain trials, for example, for the low probability stop block the instruction said: “Take a break! During the next block, stop trials will occur on one third of the uncertain go trials (yellow circle)” All instructions were in black text except for the words “one third” which were colored red and the word “yellow” which was colored yellow. Participants pressed the space bar to start the block at which point the block number was presented for 1 s, e.g., “Block 1 out of 6.” In total there were 12 blocks, 6 were assigned to vocal and 6 to manual responses. All 6 blocks per response modality were administered sequentially. After that, the other 6 blocks of other response modality were given.

The index of reactive inhibition was measured with the stop-signal reaction time or SSRT (Logan and Cowan, 1984) and calculated using the integration method (Verbruggen and Logan, 2009a; Verbruggen et al., 2013). This method estimates SSRTs by subtracting the starting time of the stop process (when participants see a stop-signal) from the finishing time of the stop process. The starting time is known, which is equivalent to the SSD; however, the finishing time needs to be estimated. The finishing time was estimated by integrating the go reaction time (go RT) distribution. The go RTs were rank ordered from the shortest to the longest then, the *nth* RT was selected. Where *n* was obtained by multiplying the probability of responding on stop trials (or unsuccessful stopping, known as the p_respond) by the total number of go RTs. The probability of responding was calculated as the number of unsuccessful stops divided by the total number of stop trials. SSRT was estimated by subtracting the SSD from *nth* go RT. We calculated the SSRT separately for each block and then the average of the blocks was taken as the final SSRT.

The index of proactive inhibition was based on two previous studies (Chikazoe et al., 2009; Jahfari et al., 2010) where it was respectively termed preparation cost and response delay effect. This index is estimated from the go RT by subtracting the mean of the uncertain go RTs from the mean of the certain go RTs. A positive value indicates the amount of slowing the participants applied to their go responses when stop signals were imminent.

All correlations were obtained from a Pearson's linear correlation (1-tailed as the proposed hypotheses were unidirectional).

### Impulsivity inventory

We administered a version of Dickman's impulsivity inventory (Dickman, 1990) which measures functional and dysfunctional impulsivity. Dysfunctional impulsivity is defined as the tendency to act with less forethought than most people of same ability when this inclination is a source of difficulty. In contrast, functional impulsivity is the tendency to act with relatively little forethought when such a style is optimal. This inventory has 46 questions: 11 about functional impulsivity, 12 for dysfunctional impulsivity and 23 fillers, which were not included in any statistical analysis (see Appendix 1 in Supplementary Material).

### Procedure

We first introduced the task verbally by explaining the three types of trials and the types of responses subjects should give. Then, the experimenter read a colored photocopy with a diagram of the trials and response types. We explained that the time between the stop-signal and the uncertain go signal (i.e., SSD) changed according to the participant's performance and that if they successfully stopped, the next stop trial would be harder because the SSD was going to be longer. We also explained that if they failed to stop, next stop trial would be easier because the SSD was going to be shorter and it would be easier for them to stop. We told them that they would fail on about 50% of stop trials because the experimental program was designed to find the balance between the ability to stop and not and therefore, they should not feel frustrated if they were not able to successfully stop on all trials. We explained that both tasks (i.e., going and stopping) were equally important and they should learn a trade-off between them. After this, we proceeded to do a practice task, which contained 6 blocks with 18 trials in each block. Once again the experimenter read the instructions out from the computer screen and additionally included information about the warning buzz; the experimenter explained that this would help them to gauge their performance and to guide them if any of their main tasks required more attention. They would hear a buzz when a response was too slow indicating that they needed to react faster on the next trial. We explained that this buzz could occur after certain or uncertain go trials. We also explained that they may hear a buzz after a stop trial and this would mean that they had failed to stop too many times and therefore needed to put extra effort into stopping successfully.

## Results

### Testing the assumptions of the horse race model

As described in the horse race model (Logan and Cowan, 1984), independence of go and stop processes is assumed because failed stop RTs are faster than the no signal RT. In this experiment we had two RTs, one RT from the uncertain go and the other from the certain go, therefore, we included these two go RTs in the analysis of independence. We conducted a repeated-measures 2 × 2 × 3 ANOVA with within-subject factors of 2 response modalities (manual, vocal), 2 stop probabilities (high and low), and 3 RT types (certain go, uncertain go and failed stop). The results revealed that all three factors were significant: response modality [*F*_(1, 43)_ = 46, *p* < 0.0001, ŋ^2^_*p*_ = 0.52], stop probability [*F*_(1, 43)_ = 1.2, *p* < 0.0001, ŋ^2^_*p*_ = 0.82] and RT type [*F*_(2, 86)_ = 83, *p* < 0.0001, ŋ^2^_*p*_ = 0.66]. The interaction between stop probability and RT was also statistically significant [*F*_(2, 86)_ = 1.2, *p* < 0.0001, ŋ^2^_*p*_ = 0.77]. All other interactions were non-significant. Because the significant interaction of stop probability and RT type confounds the main effect of these two factors we proceed to describe only this interaction and the response modality main effect.

The response modality factor showed that RTs of the manual responses (*M* = 411 ms, *SE* = 10) were 54 ms earlier compared to those of the vocal responses (*M* = 465 ms, *SE* = 10). The interaction of stop probability and RT showed that all RTs from the high probability stop were statistically longer compared to those of the low probability stop (*p* < 0.001 Bonferroni corrected, see Appendix 3 in Supplementary Material for mean and *SE*) by 20, 112, and 44 ms in the certain go, uncertain go and failed stop trials respectively. Across RTs, this interaction showed that failed stop RTs were significantly shorter than for uncertain go trials (*p* < 0.001) by 128 and 60 ms in the high and low probability stop conditions respectively. This result confirmed the assumption of the horse race model in which failed stop RTs should be faster than no stop signal RTs, suggesting the go process won the race against the stop process in the failed stop condition. Interestingly, failed stopping was not different from certain go, *p* = 0.42 and 0.67 in the high and low probability stops respectively. This finding also supports the assumptions of the horse race model in which failed stop RTs are not different from simple RT (described in more detail in the Discussion).

### Go reaction times

A repeated measures 2 × 4 ANOVA was conducted for go RTs that contained the within-subject factors of two response modalities (manual, vocal) and four go-certainty types (certain go_-*Low-probability-stop*_, certain go_-*High-probability-stop*_, uncertain go_-*Low-probability-stop*_, and uncertain go_-*High-probability-stop*_). Both factors, response modality [*F*_(1, 43)_ = 44, *p* < 0.0001, ŋ^2^_*p*_ = 0.5] and go-certainty type [*F*_(3, 129)_ = 102, *p* < 0.0001, ŋ^2^_*p*_ = 0.7] were statistically significant with a large effect size. The response modality by go-certainty type interaction was not significant [*F*_(3, 129)_ = 0.3, *p* = 0.8, ŋ^2^_*p*_ = 0.006].

**Figure 2:**
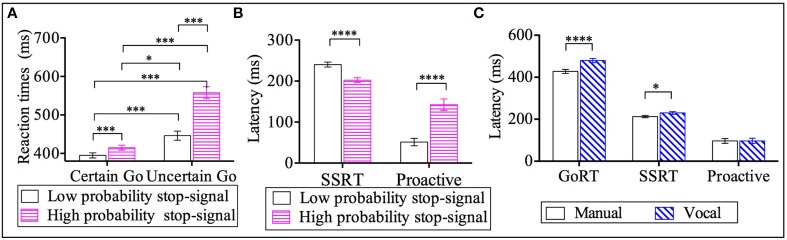
**Results for go reaction times (go RT), stop signal reaction time (SSRT) and proactive inhibition across response modalities (manual and vocal) and stop signal probabilities: high and low [2/3 stop and 1/3 stop trials following the uncertain go]. (A)** Go reaction times for the two types of go RT (certain go and uncertain go) across the two stop probabilities (low and high stop probabilities). **(B)** SSRT and proactive inhibition as a function of stop signal probability. **(C)** Latencies for go-RT, SSRT and proactive inhibition across response modalities. ^*^p < 0.05; ^***^p < 0.001; ^****^p < 0.0001. Error bars indicate standard error of the mean (SEM).

The factor of response modality (see Figure 2C) showed that go RTs from the manual responses (*M* = 428 ms, *SE* = 9) were shorter compared to vocal responses by 51 ms (*M* = 479 ms, *SE* = 10). Furthermore, the factor of go-certainty type (see Figure 2A) revealed that go RTs from the certain go_-*Low-probability-stop*_ (*M* = 395 ms, *SE* = 6.6) were significantly shorter: by 20 ms relative to certain go_-*High-probability-stop*_ (*M* = 415 ms, *SE* = 6, *p* < 0.001), by 51 ms compared to uncertain go_-*Low-probability-stop*_ (*M* = 446 ms, *SE* = 12, *p* < 0.001) and by 163 ms compared to uncertain go_-*High-probability-stop*_ (*M* = 558 ms, *SE* = 16, *p* < 0.001). The go RTs from the certain go_-*High-probability-stop*_were also significantly shorter by 31 ms compared to uncertain go_-*Low-probability-stop*_ (*p* < 0.05) and by 143 ms compared to uncertain go_-*High-probability-stop*_ (*p* < 0.001). Finally, the uncertain go_-*Low-probability-stop*_ was significantly shorter compared to the uncertain go_-*High-probability-stop*_ by 112 ms.

### Reactive inhibition (SSRT)

A repeated measures 2 × 2 ANOVA was carried out for SSRTs with the within-subject factors of two response modalities (manual, vocal) and two stop probabilities(high and low probability stop). Both factors, response modality [*F*_(1, 43)_ = 7, *p* < 0.05, ŋ^2^_*p*_ = 0.14] and stop probability [*F*_(1, 43)_ = 34, *p* < 0.0001, ŋ^2^_*p*_ = 0.4], were statistically significant. The response modality main effect exhibited a medium effect size and the stop probability main effect, a large effect size. The interaction of response modality and stop probabilitywas not statistically significant [*F*_(1, 43)_ = 0.003, *p* = 0.96, ŋ^2^_*p*_ = 0.001].

*Post-hoc* analysis within the response modality factor revealed that the SSRT of manual responses (*M* = 213 ms, *SE* = 6) was shorter by 17 ms relative to the SSRT of vocal responses (*M* = 230 ms, *SE* = 6); see Figure 2C. Further, the factor of stop probability showed that SSRT_-*Low-probability-stop*_ (*M* = 240 ms, *SE* = 6) was 38 ms longer compared to the SSRT_-*High-probability-stop*_ (*M* = 202 ms, *SE* = 6); see Figure 2B.

### Analyses of SSD and accuracy of stopping

Because we obtained a significant difference in SSRT between vocal and manual responses, we wanted to make sure this difference was not driven by the difference in go RTs: vocal responses compared to manual responses had longer go RTs. We conducted repeated measures 2 × 2 ANOVA separately for SSD and accuracy of stopping. We included the within-subject factors of 2 response modalities (manual, vocal) and 2 stop probabilities (high and low probability stop).

The results for the SSD revealed a significant difference in the factors of response modality [*F*_(1, 43)_ = 7.05, *p* < 0.05, ŋ^2^_*p*_ = 0.15] and stop probability [*F*_(1, 43)_ = 1.6, *p* < 0.01, ŋ^2^_*p*_ = 0.76]. The interaction of response modality by stop probability was not significant [*F*_(1, 43)_ = 1.49, *p* = 0.23, ŋ^2^_*p*_ = 0.04]. The response modality factor showed that SSD of the vocal responses (*M* = 265 ms, *SE* = 16) were shorter by 38 ms compared to those of the manual responses (*M* = 303 ms, *SE* = 18). In addition, the stop probability factor revealed that the SSD of the high probability stops (*M* = 333 ms, *SE* = 17.3) were 99 ms longer than those of the low probability stops (*M* = 234 ms, *SE* = 14).

As for the results of accuracy of stopping, with the staircase procedure, we expected a probability of successful stops and failed stops of about 50% each (p_inhibit = 0.5; p_respond = 0.5). The ANOVA revealed a significant factor of stop probability [*F*_(1, 43)_ = 79.71, *p* < 0.01, ŋ^2^_*p*_ = 0.65], which revealed that participants stopped slightly more successfully in the high probability stop (p_inhibit = 0.52, *SE* = 0.005) relative to the low probability stop (p_inhibit = 0.49, *SE* = 0.005). There were no statistically significant effects of response modality [*F*_(1, 43)_ = 0.93, *p* = 0.35, ŋ^2^_*p*_ = 0.03] or significant interaction between response modality and stop probability [*F*_(1, 43)_ = 1.49, *p* = 0.23, ŋ^2^_*p*_ = 0.04]. For the response modality factor, both manual and vocal responses had a p_inhibit of 0.51.

In sum, the results of the SSD and accuracy of stopping analyses suggest that the differences in SSRT between response modalities are not driven by the longer go RT as across response modalities the SSDs were also significantly different and the accuracy of stopping was statistically the same.

### Proactive inhibition

A repeated measures 2 × 2 ANOVA was conducted for proactive inhibition with within-subject factors of 2 response modalities (manual, vocal) and 2 stop probabilities (high and low probability stop). The results revealed a significant effect of stop probability [*F*_(1, 43)_ = 1.9, *p* < 0.0001, ŋ^2^_*p*_ = 0.8]. No significant effects of response modality [*F*_(1, 43)_ = 0.001, *p* = 0.97, ŋ^2^_*p*_ = 0.0001] or significant interaction between response modality and stop probability [*F*_(1, 43)_ = 1.4, *p* = 0.25, ŋ^2^_*p*_ = 0.03] were found.

The significant effect of stop probability showed that proactive inhibition for low probability stops (*M* = 51 ms, *SE* = 9) was 91 ms significantly shorter compared to the high probability stops(*M* = 142 ms, *SE* = 14); see Figure 2B.

### Correlations between reactive and proactive inhibitions

**Figure 3:**
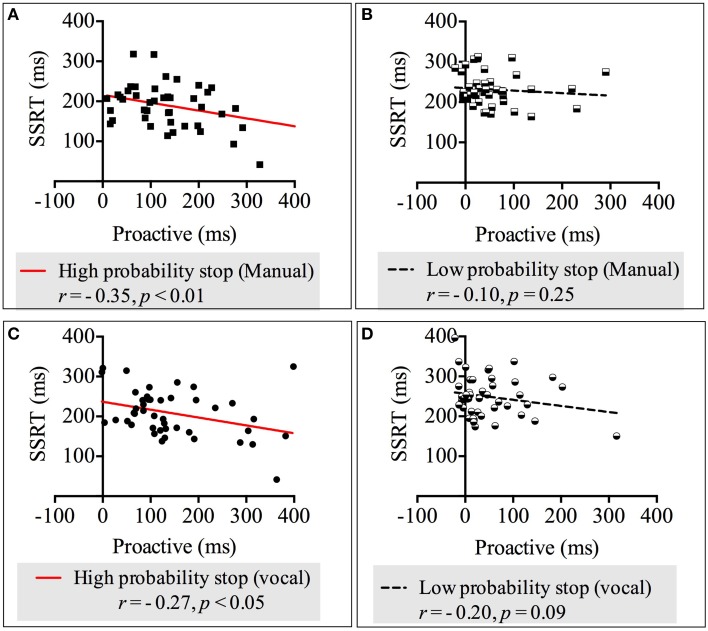
**Correlations between proactive and reactive inhibition across response modalities (manual and vocal) and stop probabilities: high and low [2/3 and 1/3 stop trials following the uncertain go]. (A)** Correlation between proactive inhibition and SSRT in the high probability stop condition for manual responses. **(B)** Correlation between proactive inhibition and SSRT in the low probability stop condition for manual responses. **(C)** Correlation between proactive inhibition and SSRT in the high probability stop condition for vocal responses. **(D)** Correlation between proactive inhibition and SSRT in the low probability stop condition for vocal responses. Because our alternative hypothesis was in one direction, all Pearson's correlations tested significance with a 1-tailed test.

We carried out four correlation analyses between reactive (measured by the SSRT) and proactive inhibition across both response modalities and stop probabilities. The results showed there were moderate negative, statistically significant relationships between proactive_-*High-probability-stop*_ and SSRT_-*High-probability-stop*_ in both response modalities: vocal [*r*_(42)_ = −0.35, *p* < 0.01] and manual [*r*_(42)_ = −0.27, *p* < 0.05]. These relationships revealed that more advanced preparation for stopping in the high probability stop condition was related to faster reactive stopping. See Appendix 2 in Supplementary Material for the non-significant correlations in the low probability stop conditions. Figure 3 depicts the correlation between the SSRT and proactive inhibition.

### Correlation between reactive inhibition and impulsivity

**Figure 4:**
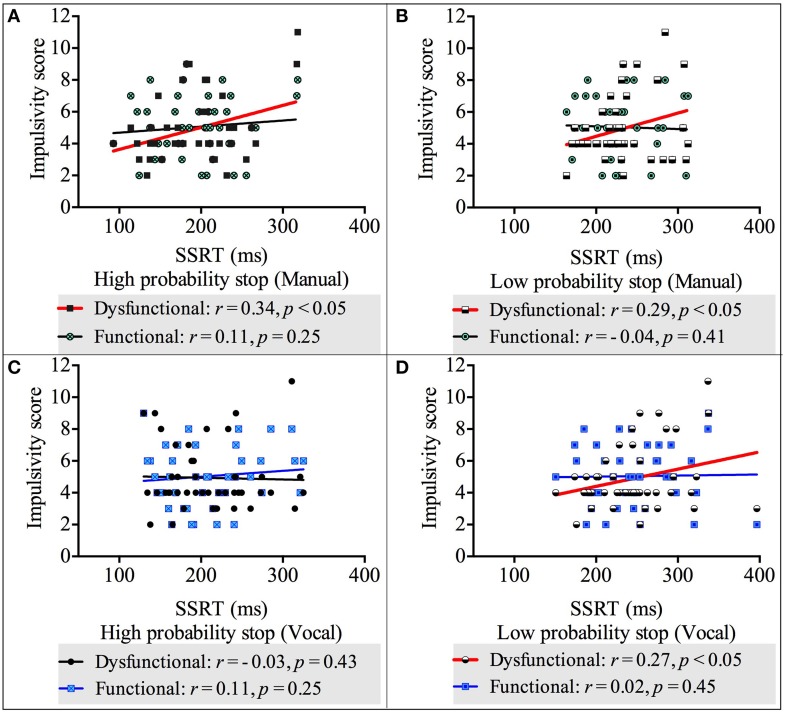
**Correlations between impulsivity score and reactive inhibition across response modalities (vocal and manual) and stop probabilities: high and low [2/3 and 1/3 stop trials followed the uncertain go]. (A)** Correlation between impulsivity score and SSRT in the high probability stop condition for manual responses. **(B)** Correlation between impulsivity score and SSRT in the low probability stop condition for manual responses. **(C)** Correlation between impulsivity score and SSRT in the high probability stop condition for vocal responses. **(D)** Correlation between impulsivity score and SSRT in the low probability stop condition for vocal responses. Because our alternative hypothesis was in one direction, all Pearson's correlations tested significance with a 1 tailed test.

We carried out four correlation analyses between reactive (measured by the SSRT) and impulsivity scores (both dysfunctional and functional impulsivity scores) across both response modalities and stop probabilities. The results showed there was a positive, statistically significant relationship between dysfunctional impulsivity and the SSRT_-*High-probability-stop*_ for manual responses [*r*_(42)_ = 0.34, *p* < 0.05]. Likewise, there were positive, statistically significant relationships between dysfunctional impulsivity and the SSRT_-*Low-probability-stop*_ for manual responses [*r*_(42)_ = 0.29, *p* < 0.05] and for vocal respones [*r*_(42)_ = 0.27, *p* < 0.05]. These relationships revealed that higher scores of dysfunctional impulsivity are related to slower reactive inhibition. See Appendix 2 in Supplementary Material for the non-significant correlations between the SSRT_-*High-probability-stop*_ and dysfunctional impulsivity;and between the SSRTs (both high and low probability stops) and functional impulsivity. See Figure 4 for a graphical representation of the correlations between the SSRT and impulsivity.

### Correlation between proactive inhibition and impulsivity

We carried out 4 correlations in each response modality. These correlations compared the index of proactive inhibition across stop probabilities (high and low probability stops) with scores on impulsivity scales (functional and dysfunctional scores). All correlations were non-significant, the results of these correlations can be found Appendix 2 in Supplementary Material.

### Correlation between manual and vocal responses

**Figure 5:**
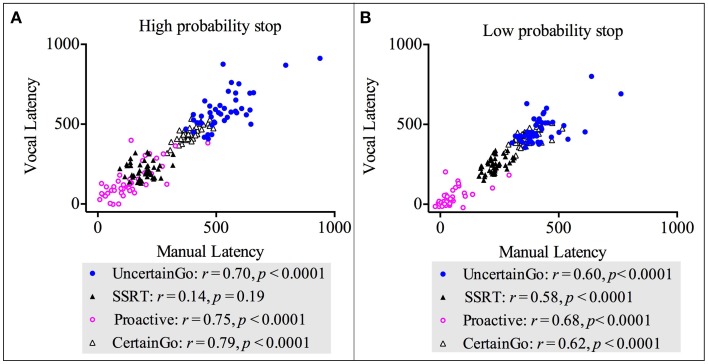
**Correlations between vocal and manual responses for both go RT types (certain and uncertain) and both inhibition types (proactive and reactive). (A)** Correlations for the high probability stop [2/3 stop trials followed the uncertain go]. **(B)** Correlations for the low probability stop [1/3 stop trials followed the uncertain go].

We conducted correlations between manual and vocal responses across both go RT types (certain go and uncertain go) and both inhibition types (reactive and proactive). The results revealed strong, positive, statistically significant relationships between manual and vocal responses in: certain go RT-_High probability stop_ [*r*_(42)_ = 0.79, *p* < 0.0001]; uncertain go RT-_High probability stop_ [*r*_(42)_ = 0.70, *p* < 0.0001]; proactive-_High probability stop_ [*r*_(42)_ = 0.75, *p* < 0.0001]; certain go RT-_Low probability stop_ [*r*_(42)_ = 0.62, *p* < 0.0001]; uncertain go RT-_Low probability stop_ [*r*_(42)_ = 0.60, *p* < 0.0001]; proactive-_Low probability stop_ [*r*_(42)_ = 0.68, *p* < 0.0001] and SSRT-_Low probability stop_ [*r*_(42)_ = 0.58, *p* < 0.0001]. These relationships suggested that when one index in vocal responses increased, the counterpart index in manual responses increased too. There was only the correlation of SSRT-_High probability stop_ between responsemodalities that was not statistically significant [*r*_(42)_ = 0.14, *p* = 0.19]. These results are depicted in Figure 5. Interestingly, the SSRT-_High probability stop_ was the only index that did not correlate with dysfunctional impulsivity.

## Discussion

This study investigated reactive and proactive response inhibition in two effector systems: vocal and manual. We also examined the relationship between these two types of response inhibition and self-reported functional and dysfunctional impulsivity. We hypothesized that conditions where stopping was required with a high probability (1/3 stops) compared to low probability (1/6 stops) stops would enhance both proactive and reactive response inhibition. Secondly, we hypothesized that reactive and proactive response inhibition would be positively related (a negative slope in the correlation between SSRT and proactive inhibition). Last, that SSRT and dysfunctional impulsivity would be positively correlated, i.e., as dysfunctional impulsivity increased, SSRTs would be slower. Our results provide evidence to support all of these hypotheses, which are discussed in turn below.

### High compared to low probability stop condition increased proactive and enhanced reactive response inhibition

As predicted, we found that in the high relative to the low probability stops, proactive inhibition was longer (by 91 ms) and SSRT was shorter (by 38 ms). The results suggest that a greater level of preparation for the impending stop signal was implemented when there was a higher probability of stopping; this preparation was also transferred to the go RT of the certain go (only go) and the uncertain go (includes go and stop trials) conditions, as go RTs were longer in the high compared to the low probability stops. These findings are consistent with the previous literature showing that go RTs are affected by stop probability, e.g., go RTs are longer in the most frequent stop signal conditions (Ramautar et al., 2004; Lansbergen et al., 2007) and even after a stop-signal, e.g., go RTs are slower after a stop trial (Rieger and Gauggel, 1999; Emeric et al., 2007; Verbruggen et al., 2008; Verbruggen and Logan, 2008a). These findings are also consistent with the proactive adjustment hypothesis (Verbruggen and Logan, 2009b) that assumes subjects balance stop and go processes by increasing the response threshold in the go task when they expect more stop signals.

Supporting our hypothesis, apart from increased proactive response inhibition, SSRTs were shorter in the high probability stop condition. Interestingly, SSRT has also been observed to be shorter in conditions in which participants are informed of the position of the stop signal compared to an uninformed condition (Smittenaar et al., 2013). This means, that more preparation induced either by a high probability stop or an informed condition enhances reactive inhibition. We also found that in the high probability stops, participants stopped successfully more often (p_inhibit 0.52 compared to 0.49 in the low probability), a finding which is in line with Ramautar et al. (2004) and suggests, participants are slightly biased toward successful inhibition over fast responding. These finding is also consistent with more recent studies that have shown that higher probability of stopping is associated with prolonged go RT, indicating higher proactive inhibition (Hu et al., 2015).

Contrary to the finding that the high probability stop enhances reactive inhibition, other studies have found that changing the stop probability has no effect on the SSRT (Logan and Burkell, 1986; Ramautar et al., 2004; Lansbergen et al., 2007). The reasons could be the small sample size in these previous studies (~13 participants) whereas our data comes from 44 participants. Another reason could be the percentage of the high probability stops, for example, the high probability stop condition in Ramautar et al. (2004) was 50% of stops and 50% of go, whereas in our study the stops in the high probability were 66.66% of uncertain go, potentially eliciting more preparation. Another reason for the differences could be the amount of stops, while we had 100 stops for each stop probability, Logan and Burkell (1986) had 48 stops in the low probability stop against 192 stops in the high probability stop. Finally, Lansbergen et al. (2007) study was comparing differences in impulsivity, thus, the participants recruited were 14 with the lowest scores in impulsivity and 15 with the highest scores in impulsivity. This could have made the results different to our study. In short, the different findings we present in this study compared to those previous studies (Logan and Burkell, 1986; Ramautar et al., 2004; Lansbergen et al., 2007) could be related to the differences in stop probability distributions, sample size, stop trial size and the particular characteristics of the sample.

The assumption of the horse race model, in which failed stop RTs should be faster than go RTs, was met in both stop probability conditions, in line with previous studies (Logan and Cowan, 1984; Logan, 1994; Ramautar et al., 2004; Chikazoe et al., 2009). On the other hand, the finding that SSRTs are different across stop probabilities suggests that the stopping process is not constant, an assumption that agrees with the independent horse race model. It is worth noting that the independent horse race model estimates the SSRT assuming “*the finishing time of the stopping process (stop signal reaction time) is constant”* (Logan, 1994); “…*the assumption about stop signal reaction time makes mathematics easier, but more importantly, it allows a graphic representation of the underlying processes that illustrates the relationships very clearly”* (Logan, 1994); “*the correctness of the assumption is not very important*. Logan and Cowan (1984) *(mostly Cowan) analyzed the formal consequences of the assumption, and found that it introduced very small measurement errors”* (Logan, 1994).

We conclude that greater levels of preparation, represented by increased proactive inhibition in the high stop probability, reduced the time of reactively stopping a prepotent response. We further supported this idea with our second hypothesis, which is described next.

### Reactive and proactive response inhibition have a positive relationship

Our second hypothesis measured the relationship between reactive and proactive inhibition. We found that a greater level of preparation was related to reduced SSRT. This was only observed for the high probability stops in both response modalities. These findings support two out of three experiments in Chikazoe et al. (2009) and Jahfari et al. (2010). Although the amount of stops was very similar in these two studies (20% of stops in the uncertain go condition and 25% of stops respectively) to our low probability stop condition (33.33% of stops in the uncertain go), we did not find that reactive and proactive were negatively related in the low probability stops, like one experiment in Jahfari et al. (2010). We suggest that this could have been because participants, in the two previous experiments that found a relationship, applied more proactive inhibition compared to the low probability stops. In fact, Jahfari et al. (2010) carried out two experiments and only found that reactive and proactive inhibition were related in experiment 1 where proactive inhibition was larger (111.3 ms in all trials) compared to experiment 3 in which proactive inhibition was much smaller (55 ms). Similarly, Chikazoe et al. (2009) found that reactive and proactive inhibition were related when proactive inhibition was 105.5 ms. In our study, the high probability stop condition elicited proactive slowing of 142 ms compared to the low probability stop condition where proactive slowing was only 51 ms. These results suggest that when participants used greater level of preparation to hold the prepotent response (proactive inhibition), they could stop faster, but that when they did not withhold the prepotent response strongly, reactive inhibition was executed by other process not related to the amount of proactive inhibition.

Taken together (hypotheses one and two), we conclude that our findings are consistent with the proactive adjustment account (Verbruggen and Logan, 2009b) in which participants balance stop and go processes by increasing the go RT when the stop probability increases. These results also support the account that greater level of proactive inhibition enhances reactive inhibition. It could be that this alert to hold the prepotent response pre-activates some of the same neural circuitry responsible for reactive inhibition but only when it is very likely that stopping will occur, as described in Aron (2011). In short, it seems that the go process and reactive inhibition interact with proactive inhibition (Boucher et al., 2007; Verbruggen and Logan, 2008b).

### Relationship between reactive inhibition and dysfunctional impulsivity

Our last hypothesis predicted that there would be a positive relationship between the SSRT and dysfunctional impulsivity. This is what we found, that slower SSRT (weaker reactive stopping) was related with a higher score on dysfunctional impulsivity (not functional impulsivity). This is consistent with a previous study that used the same Dickman impulsivity inventory (Dickman, 1990) and found this relationship existed for vocal responses only (van den Wildenberg and Christoffels, 2010). We extended this relationship to manual responses, which is consistent with other studies that have tested this effector system and found that SSRTs were related to impulsivity scores (Logan et al., 1997; Marsh et al., 2002; Farr et al., 2012). This relationship between reactive inhibition and self-reported impulsivity is consistent with pathological studies that have found slower SSRTs in neuropsychological disorders where impulsive behavior is a major characteristic (Rubia et al., 2001b; Aron and Poldrack, 2005; Nigg et al., 2006; Menzies et al., 2007; Penadés et al., 2007; Bohne et al., 2008; Enticott et al., 2008). Interestingly, this relationship was only seen for reactive inhibition and not for proactive inhibition, suggesting that self-reported impulsivity is more related with overt inhibitory responses.

However, some studies have found that no relationship existed between the SSRTs and impulsivity (Avila and Parcet, 2001; Rodrìguez-Fornells et al., 2002; Lijffijt et al., 2004; Lansbergen et al., 2007). One of the reasons these studies did not find a relationship could be that they used a different impulsivity inventory; these studies used the 54 item Eysenck impulsivity scale (I7, Eysenck et al., 1985). Studies that found a relationship between SSRTs and impulsivity have used a different impulsivity inventory, for example, Logan et al. (1997) used the Eysenck Personality Inventory (Eysenck and Eysenck, 1969) which contained 22 true-false questions; the other two studies (Marsh et al., 2002; Farr et al., 2012) used the Barratt impulsiveness scale, version 11 (Barratt and Patton, 1983). It is very likely that these different impulsivity inventories are measuring distinctive dimensions of impulsivity. For example, the subscales measured in the Eysenck Personality Inventory (Eysenck and Eysenck, 1969) are impulsivity and sociability whereas the subscales measured in I7 are impulsivity, venturesomeness and empathy. Another reason could lie in the characteristics of the participants. While Logan et al. (1997) recruited students, some of the studies that did not find a relationship recruited high and low impulsive participants (Rodrìguez-Fornells et al., 2002; Lijffijt et al., 2004; Lansbergen et al., 2007). In short, the instruments to assess impulsive characteristics and the characteristics of the participants may explain why the SSRTs were not related with impulsivity scores in previous studies.

### Additional findings across response modalities

Across response modalities we found that the go RTs for vocal responses were slower compared to those of manual responses. This is consistent with previous studies (van den Wildenberg and Christoffels, 2010). Another study also found that naming part words was slower compared to both manual and naming letters (Xue et al., 2008). Other studies that have tested vocal responses but not compared them directly with manual responses showed that go RT of vocal responses is slower compared to those of manual responses (Wessel and Aron, 2015).

We also found that the SSRT were slower in vocal compared to manual responses but proactive inhibition was the same across these two response modalities. These results cannot really be explained with the proactive adjustment account that suggests larger proactive inhibition enhances reactive inhibition. Moreover, the SSDs were shorter in vocal compared to manual responses. We explained this with the hypothesis that vocal responses have less efficacious reactive inhibition. For example, neurophysiological studies have suggested that corticobulbar motoneurons (which supply some of the vocal muscles) are sparser or less potent than the spinal motoneurons (limb muscles; Jaberzadeh et al., 2008; Ortu et al., 2008; Sowman et al., 2008; for a review see Luschei and Goldberg, 2011). For example, studies investigating the cortical silent period (CSP) on the muscles of the vocalization system (cranial nerve V) describe shorter CSP compared to studies investigating the CSP in the limb system (Werhahn et al., 1995; Cruccu et al., 1997; Paradiso et al., 2005; Jaberzadeh et al., 2008; Ortu et al., 2008; Sowman et al., 2008).

Finally, we found that both go RT (certain and uncertain go), and both reactive and proactive inhibitions were positively correlated between manual and vocal responses across both stop probabilities. The only relationship across response modalities that was not significant was between the SSRT in the high probability stops. Surprisingly, the SSRT of vocal responses in the high probability stops was not related with dysfunctional impulsivity either. These results suggest that the SSRTs of the high probability stops for vocal responses behave differently to those of manual responses and does not relate with impulsivity. The only reason we could think to explain this finding is via the already described hypothesis that the vocal system has less efficacious reactive stopping. Based on this account, SSRTs of the vocal system might dissociate from those of the manual responses in the high probability stop condition where reactive stopping mechanisms are under the highest performance demand. However, further studies would be required to support this hypothesis.

## Conclusions

This study investigated response inhibition in two response modalities (i.e., manual and vocal) and related them to self-reported functional and dysfunctional impulsivity. We found that high compared to low probability stops required more proactive inhibition and produced faster reactive stopping. This was further confirmed in a correlation analysis that showed greater levels of preparation for stopping reduced the SSRT. We also showed that SSRTs were related to dysfunctional impulsivity. The implications of these findings extend the horse race model by studying proactive response inhibition, which currently encompasses only go RT and the SSRT variables. Our results show that proactive inhibition can enhance reactive inhibition by reducing the SSRT only when proactive inhibition is applied strongly. From a therapeutic point of view, these findings can help to reduce impulsive behaviors by for example, cognitive reinforcement on functional instead of dysfunctional impulsivity; in addition, cognitive training that favors increment of proactive inhibition could potentially reduce impulsive behaviors.

## Funding

This research was supported by Macquarie University Research Excellence Scholarships (MQRES), National Health and Medical Research Council, Australia (#1003760) and the Australian Research Council (DE130100868).

### Conflict of interest statement

The authors declare that the research was conducted in the absence of any commercial or financial relationships that could be construed as a potential conflict of interest.
