# Supplementary material for: The effects of impulsivity and proactive inhibition on reactive inhibition and the go process: insights from vocal and manual stop signal tasks
Source: Front Hum Neurosci. 2015 Oct 6;9:529. doi: 10.3389/fnhum.2015.00529 (PMC4594014; doi:10.3389/fnhum.2015.00529)

**Appendix 1**

Dickman’s impulsivity inventory (Dickman, 1990)

| Filler_Q1 | I would travel a great deal if I had the chance. |
| --- | --- |
| F_Q1 | I don't like to make decisions quickly, even simple decisions, such as choosing what to wear, or what to have for dinner |
| Filler_Q2 | I seldom tell lies. |
| D_Q1 | I will often say whatever comes into my head without thinking first. |
| Filler_Q3 | I have many hobbies. |
| F_Q2 | I am good at taking advantage of unexpected opportunities, where you have to do something immediately or loose your chance. |
| Filler_Q4 | I would rather read fiction than non-fiction. |
| D_Q2 | I enjoy working out problems slowly and carefully. |
| Filler_Q5 | I would not drive over the speed limit even if I knew I would not be caught. |
| F_Q3 | I am uncomfortable when I have to make up my mind rapidly. |
| Filler_Q6 | I consider myself a sympathetic person. |
| D_Q3 | I frequently make appointments without thinking about whether I will be able to keep them. |
| Filler_Q7 | I enjoy exercising. |
| F_Q4 | I like to take part in really fast-paced conversations, where you don't have much time to think before you speak. |
| Filler_Q8 | I like most of the people I meet. |
| D_Q4 | I frequently buy things without thinking about whether or not I can really afford them. |
| Filler_Q9 | I watch television as much as most people do. |
| F_Q5 | Most of the time, I can put my thoughts into words very rapidly. |
| Filler_Q10 | I enjoy outdoor activities. |
| D_Q5 | I often make up my mind without taking the time to consider the situation from all angles. |
| Filler_Q11 | I have read more books than most of my friends. |
| F_Q6 | I don't like to do things quickly, even when I am doing something that is not very difficult. |
| Filler_Q12 | I am more alert than most people late at night. |
| D_Q6 | Often, I don't spend enough time thinking over a situation before I act. |
| Filler_Q13 | I like to read about scientific research. |
| F_Q7 | I would enjoy working at a job that required me to make a lot of split-second decisions. |
| Filler_Q14 | Religion is very important in my life. |
| D_Q7 | I often get into trouble because I don't think before I act. |
| Filler_Q15 | I have more curiosity than most people. |
| F_Q8 | I like sports and games in which you have to choose your next move very quickly. |
| Filler_Q16 | I read the newspaper almost every day. |
| D_Q8 | Many times the plans I make don't work out because I haven't gone over them carefully enough in advance. |
| Filler_Q17 | I sometimes get depressed for no good reason. |
| F_Q9 | People have admired me because I can think quickly |
| Filler_Q18 | I enjoy it when I get a chance to visit a city I've never seen before. |
| D_Q9 | I rarely get involved in projects without first considering the potential problems. |
| Filler_Q19 | I am easily embarrassed. |
| F_Q10 | I have often missed out on opportunities because I couldn't make up my mind fast enough |
| Filler_Q20 | I am more alert than most people in the morning. |
| D_Q10 | Before making any important decisions, I carefully weigh the pros and cons. |
| Filler_Q21 | I make an effort to take care of my health. |
| F_Q11 | I try to avoid activities where you have to act without much time to think first |
| Filler_Q22 | I generally go to bed at a later hour than most people do. |
| D_Q11 | I am good at careful reasoning. |
| Filler_Q23 | I think that I am more creative than most of my friends. |
| D_Q12 | I often say and do things without considering the consequences. |

F= Functional impulsivity; D= Dysfunctional impulsivity. Q= question

**Appendix 2**

**^Table 1:^** ^Pearson’s correlations between proactive and reactive inhibition and, between both impulsivity scales (functional and dysfunctional) and both inhibition types (proactive and reactive inhibition).^

| Variables | (*r*) | (*p)* |
| --- | --- | --- |
| Proactive_-High-probability-stop (Manual)_ & SSRT_-High-probability-stop (Manual)_  Proactive_-Low-probability-stop (Manual)_ & SSRT_-Low-probability-stop (Manual)_  Proactive_-High-probability-stop (Vocal)_ & SSRT_-High-probability-stop (Vocal)_  Proactive_-Low-probability-stop (Vocal)_ & SSRT_-Low-probability-stop (Vocal)_ | - 0.16 | 0.15 |
|  | - 0.10 | 0.25 |
|  | - 0.27 | < 0.05 |
|  | - 0.20 | 0.09 |
| Dysfunctional impulsivity & SSRT_-High-probability-stop (Manual)_  Dysfunctional impulsivity & SSRT_-Low-probability-stop (Manual)_  Dysfunctional impulsivity & SSRT_-High-probability-stop (Vocal)_  Dysfunctional impulsivity & SSRT_-Low-probability-stop (Vocal)_ | 0.34 | < 0.05 |
|  | 0.29 | < 0.05 |
|  | - 0.03 | 0.43 |
|  | 0.27 | < 0.05 |
| Functional impulsivity & SSRT_-High-probability-stop (Manual)_  Functional impulsivity & SSRT_-Low-probability-stop (Manual)_  Functional impulsivity & SSRT_-High-probability-stop (Vocal)_  Functional impulsivity & SSRT_-Low-probability-stop (Vocal)_ | 0.11 | 0.25 |
|  | -0.04 | 0.41 |
|  | 0.11 | 0.25 |
|  | 0.02 | 0.45 |
| Dysfunctional impulsivity & Proactive_-High-probability-stop (Manual)_  Dysfunctional impulsivity & Proactive _-Low-probability-stop (Manual)_  Dysfunctional impulsivity & Proactive _-High-probability-stop (Vocal)_  Dysfunctional impulsivity & Proactive _-Low-probability-stop (Vocal)_ | 0.12 | 0.22 |
|  | 0.11 | 0.25 |
|  | 0.07 | 0.33 |
|  | 0.08 | 0.32 |
| Functional impulsivity & Proactive _-High-probability-stop (Manual)_  Functional impulsivity & Proactive _-Low-probability-stop (Manual)_  Functional impulsivity & Proactive _-High-probability-stop (Vocal)_  Functional impulsivity & Proactive _-Low-probability-stop (Vocal)_ | 0.05 | 0.39 |
|  | -0.01 | 0.48 |
|  | 0.04 | 0.40 |
|  | -0.03 | 0.44 |

^* Note that the SSRT were calculated from only the uncertain condition (uncertain go + stop trials). All degrees of freedom are 42.^ *^r^* ^= correlation coefficient;^ *^p^* ^= significance level, 1 tailed.^

**Appendix 3**

**^Table 2:^** ^Reaction times of certain go, uncertain go and failed stops^

| Variable | Mean (SE) |
| --- | --- |
| HP-Certain Go  HP-Uncertain Go  HP-Failed stop | 415 (7) |
|  | 558 (16) |
|  | 430 (13) |
| LP-Certain Go  LP-Uncertain Go  LP-Failed stop | 395 (7) |
|  | 446 (12) |
|  | 386 (10) |

^Variables of the interaction stop probability by RT during 2 x 2 x 3 ANOVA. HP = high probability stop; LP = low probability stop.^

**Appendix 4**

Correlations across the high and low probability stop between uncertain go with SSD and SSRT. M = manual; V = vocal


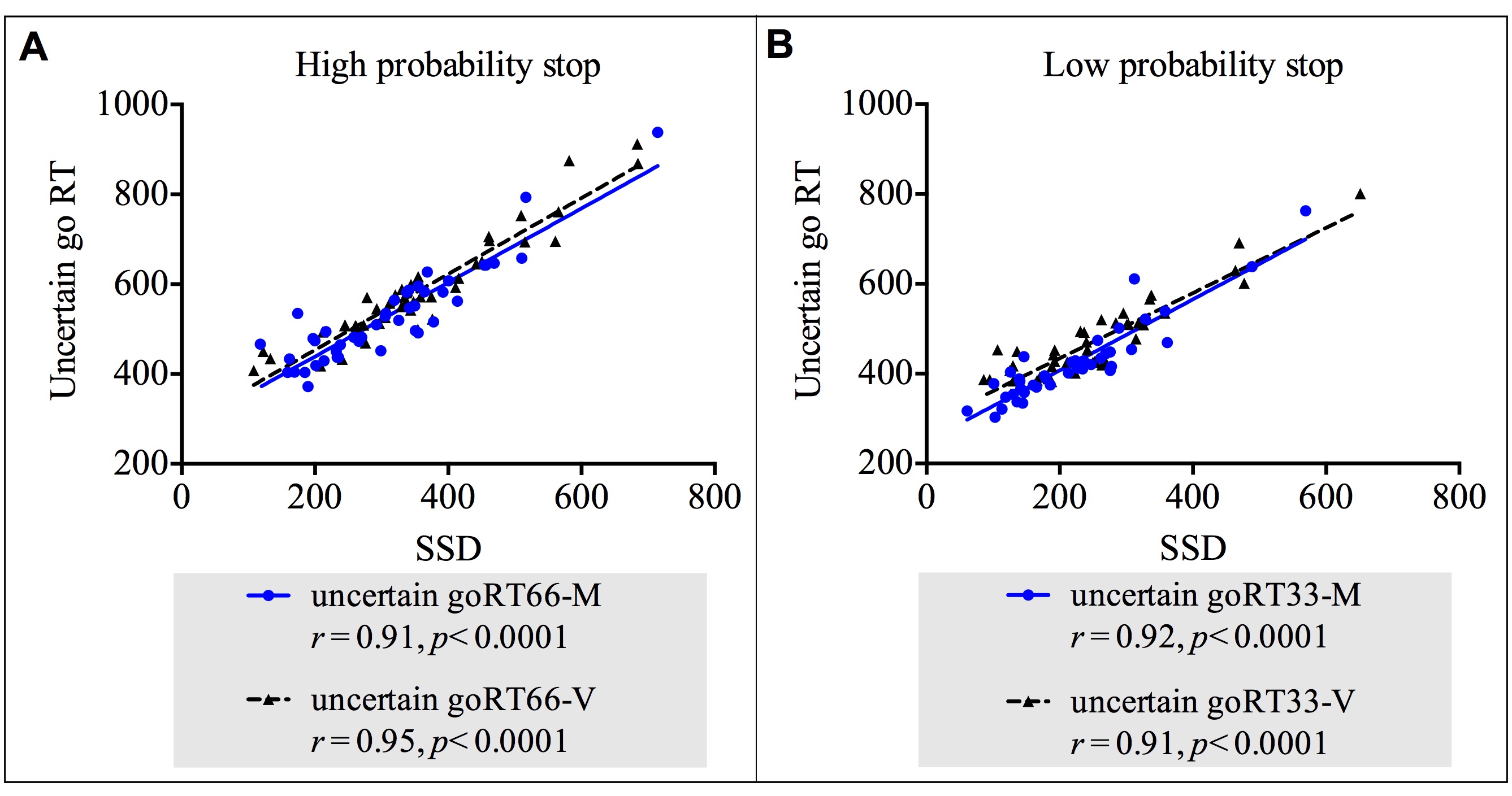


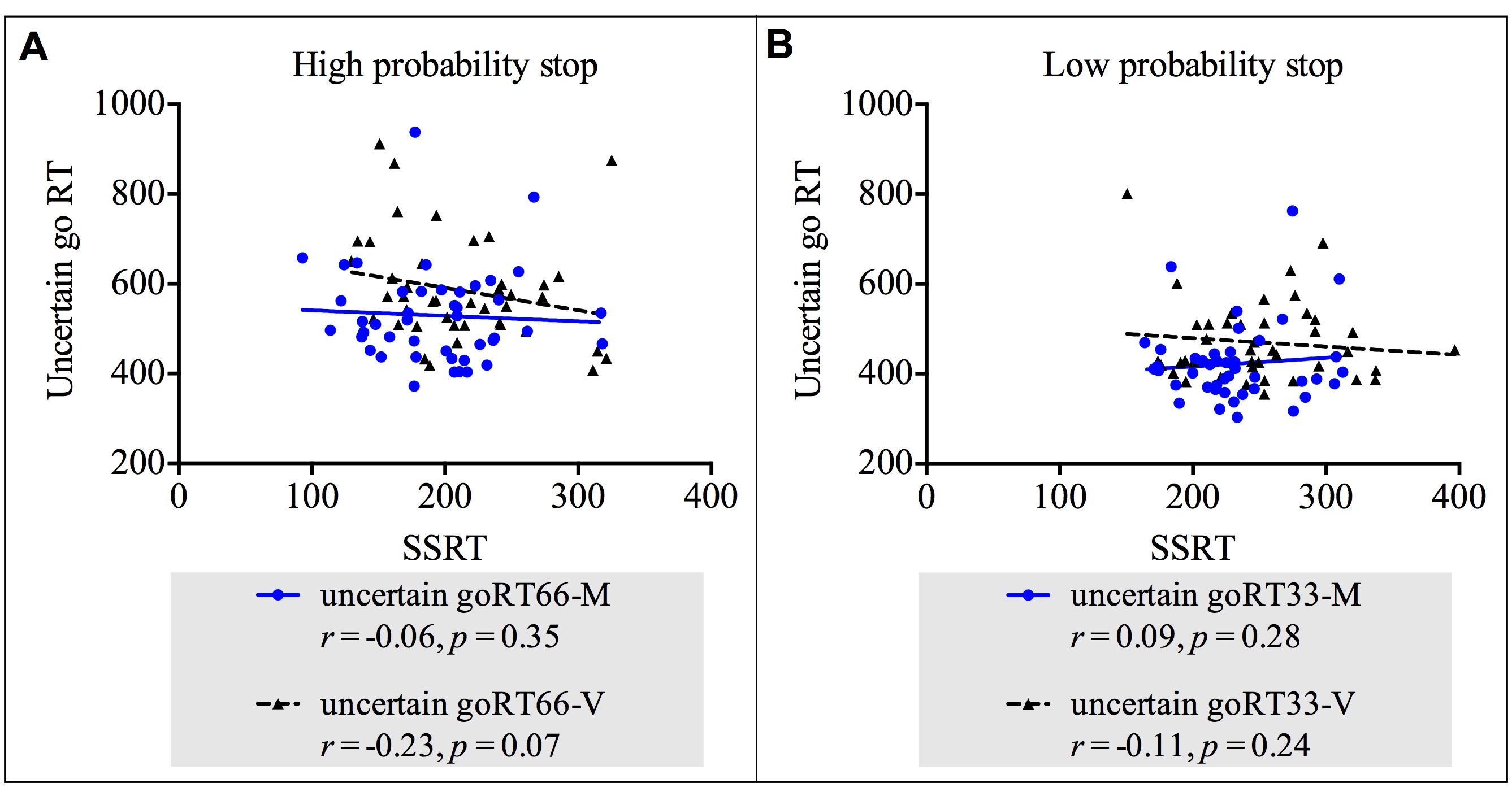

Supplement: Supplementary file 1 [file DataSheet1.DOCX]
